# Study of oxaliplatin penetration into ovaries of patients treated with hyperthermic intraperitoneal chemotherapy (HIPEC) for peritoneal metastases of colorectal and appendiceal origin using mass spectrometry imaging

**DOI:** 10.1515/pp-2020-0149

**Published:** 2021-03-24

**Authors:** Marion Larroque, Sandra Mounicou, Olivia Sgarbura, Carine Arnaudguilhem, Lucie Rebel, Cristina Leaha, Pierre-Arnaud Faye, Christine Enjalbal, François Quénet, Brice Bouyssiere, Sébastien Carrere

**Affiliations:** Institut du Cancer de Montpellier, Unité de Recherche Translationnelle, Montpellier, France; IRCM, INSERM, Univ Montpellier, Montpellier, France; Universite de Pau et des Pays de l’Adour, E2S UPPA, CNRS, IPREM, Pau, France; Département Chirurgie Oncologique, Institut du Cancer de Montpellier, Montpellier, France; Département d’Anatomopathologie, Institut du Cancer de Montpellier, Montpellier, France; Centre des Ressources Biologiques, Institut du Cancer de Montpellier, Montpellier, France; IBMM, Univ Montpellier, CNRS, ENSCM, Montpellier, France

**Keywords:** colorectal cancer, fertility preservation, low-grade appendiceal mucinous neoplasm (LAMN), mass spectrometry imaging, oxaliplatin, peritoneal cancer

## Abstract

**Objectives:**

Platinum salts are commonly used in hyperthermic intraperitoneal chemotherapy (HIPEC) for digestive tract cancer treatment. During HIPEC with oxaliplatin for peritoneal metastases (PMs) treatment, the ovaries are directly exposed to the drug, questioning about ovarian resection and the potential impact of the drug on ovarian functionality, especially in young women of childbearing age. The goal of this work is to understand unwanted damages to the ovaries during HIPEC therapy by the determination of the concentration and distribution of platinum in ovaries in order to address its potential toxicity.

**Methods:**

Mass spectrometry imaging techniques, matrix-assisted laser desorption ionization mass spectrometry (MALDI-MS) and laser ablation inductively coupled plasma mass spectrometry (LA-ICP MS), were used to study the penetration of oxaliplatin in ovaries after HIPEC treatment.

**Results:**

MALDI-MS allowed the localization of an oxaliplatin-derivative (*m/z* 456.2) at the periphery of the ovaries. The quantitative LA-ICP MS maps confirmed the localization of elemental platinum as well as in the central part of ovaries from patients who received a previous platinum salt-based chemotherapy.

**Conclusions:**

LA-ICP MS images showed that platinum diffusion was extended in cases of previous systemic treatment, questioning about platinum derivatives gonado-toxicity when combining the two treatments.

## Introduction

Peritoneal metastases (PMs) represent a frequent extension pathway of digestive cancers. Colorectal and appendicular neoplasias are frequent primary sites in Western populations. PMs are a frequent extension of colorectal cancer (CRC) (15%) due to the dissemination of tumoral digestive tract cells through the peritoneum. Before the 1980s, the treatment of PM primarily consisted of palliative management using systemic chemotherapy. From the 1990s, cytoreductive surgery supplemented with hyperthermic intraperitoneal chemotherapy (HIPEC) [1] offered a survival benefit and potential access to a cure in select patients [2].

Maximal cytoreductive surgery (CRS) followed by HIPEC has become the standard of care for pseudomyxoma peritonei (PMP) and an option for digestive tract PM [3]. The recent results of the Prodige7 trial [4] on CRC treatments stressed the role of complete surgery but diminished the frequency of indications for HIPEC. Nevertheless, HIPEC remains an option depending on the time of the diagnosis, the extent and subtype of the disease [5] and the patient’s geographical location [6].

PMs are mostly diagnosed in patients older than 55 years, but these tumors also affect young female patients who wish to have children. Therefore, the preservation of their fertility must be considered. Recent therapeutic advances significantly improved the prognosis of these patients [7] and facilitate the re-examination of the question of fertility. Advances in medically assisted procreation in oncology are a popular topic [8], [9], [10]. The actual surgical procedure for PM from the digestive tract in women recommends bilateral adnexectomy, which leaves scarce options for pregnancy because ovarian metastases are present in 52% of patients with CRS for peritoneal carcinomatosis of colorectal origin [11], [12].

Rare cases of spontaneous pregnancies were reported after peritoneal malignancies and were treated with CRS and intraperitoneal (IP) cisplatin [13] or HIPEC with cisplatin or mitomycin C [14], [15].

Oxaliplatin (derived from platinum salts) is currently a molecule of choice in the treatment of CRC. Apart from the FOLFOX protocol, oxaliplatine is also used in triplets or doublets with targeted agents in the induction treatment of metastatic colorectal disease [16], [17], [18]. This efficacy promoted it as a first-choice drug for HIPEC in PM of colorectal [19] or gastric [20] origin, and it is one of the prominent options for PMP [21].

Intravenous oxaliplatin showed a moderate risk of gonadotoxicity via alteration of follicular maturation and reduction in the reserve of primordial follicles [22]. However, the diffusion of oxaliplatin into the ovary during HIPEC was not investigated and, in regard of the enhanced cytotoxic effect of OX-HIPEC on PM [23] we hypothesize that this cytotoxic effect could also be deleterious for ovarian functions.

We previously used sensitive and resolutive imaging technologies, such as matrix-assisted laser desorption ionization mass spectrometry imaging (MALDI-MS) alone [24] or in combination with laser ablation inductively coupled plasma mass spectrometry imaging (LA-ICP MS) [25] and evaluated the penetration of platinum drugs into samples of PM of colorectal origin.

The present study localized and quantified the amount of complexed and metallic platinum (Pt) present in the ovaries of patients before and after HIPEC for PM of CRC origin using MALDI-MS and LA-ICP MS imaging. This study even recruiting post-menopausal women will allow us to get valuable information and the ethical background necessary to engage further studies on ovaries of pre-menopausal women that contribute to our understanding of the functional gonadotoxicity of oxaliplatin within a HIPEC protocol.

## Materials and methods

### Patient selection

The patients included in the study were women treated at the Montpellier Cancer Institute between January 2016 and December 2017 for a cancer of digestive origin for which the treatment of the peritoneum consisted of complete cytoreductive surgery (CCR) and an oxaliplatin-based HIPEC in the case neoplasia of appendiceal and colorectal origin. Patients were treated with an oxaliplatin-based systemic chemotherapy months before HIPEC. Morphological and macroscopic examination of the ovaries revealed no extraperitoneal or metastatic ovarian disease. Case selection was based on the principle that each patient was her own control. Patients with one or no ovaries were excluded. For this prospective study, all patients gave written informed consent before the procedure, and the Institutional Review Board approved the study, which was performed in accordance with the ethical standards of the Helsinki Declaration of 1975.

### Surgical procedures and HIPEC treatment

All patients received the standard treatment at the time for PM of digestive tract cancer origin, which consisted of CCR followed by oxaliplatin-based HIPEC using the coliseum (open) technique. As with any peritoneal surgical procedure, an exhaustive exploration of the abdominal cavity was performed to assess the extent of the disease based on the peritoneal cancer index (PCI) [26]. The macroscopic aspect of the ovaries was evaluated during this operation. If the ovaries appeared healthy macroscopically and CRS and the HIPEC procedure were feasible, then one ovary was removed before IP oxaliplatin treatment. The contralateral ovary was resected at the end of the HIPEC protocol. Only oxaliplatin was administered intraperitoneally at a temperature of 42 ± 1 °C throughout the abdominal cavity over 30 min at 250 or 460 mg oxaliplatin/m^2^. The choice of chemotherapy dose was based on histological type, PCI score, and comorbidities.

### Histological characterization

Ovaries resected from patients before and after HIPEC were transferred to the Department of Pathology for examination, and cross-sections of 3 mm in the central part of the ovary in its larger transverse axis were made from one-half frozen ovary for mass spectrometry imaging. The other half was fixed with paraformaldehyde before inclusion in paraffin for histological tissue staining. The same expert pathologist (CL) analyzed all pathological specimens using conventional hematoxylin-eosin-saffron stained microscopy.

### MALDI-MS imaging

Eight-micron-thin sections were created from frozen tissue using a Microm HM 550 Thermo Scientific cryotome (Microm International GmbH, Walldorf, Germany) at −18 °C for the specimen and −20 °C for the chamber. Sections were deposited onto a MALDI stainless steel support and allowed to thaw under vacuum desiccation for 1 h. Immediately before MALDI-MS analysis, an α-cyano-4-hydroxycinnamic acid (LaserBioLabs, France) matrix (5 mg ml^−1^ in CH_3_CN/H_2_O, 70/30 (V/V)) was sprayed onto the tissue section using a SunCollect SunChrom sprayer. MALDI-MS images were acquired on the RapifleX Analyzer (Bruker, Germany), and data were processed using FlexImaging software (MSI imaging, Bruker). Ovarian section imaging was performed under linear positive mode in the mass range of *m/z* 200–1,000 with a spatial resolution of 100 µm and laser intensity at 40% of full laser power. At each position of the tissue section, an averaged mass spectrum was generated from 800 consecutive laser shots.

### LA-ICP MS imaging

Eight micron-thick tissue slices were created as described for MALDI-MS imaging but deposited onto a glass slide. The surface of the slide was modified for platinum (Pt) quantification purposes. Briefly, Pt quantification was performed via the development of concentration calibration standards [27]. The limit of detection reached 0.1 ng mg^−1^ for Pt. Standards and ovary thin sections were sampled using a laser ablation system (NWR213, ESI, Freemont, CA) coupled to an ICP MS (7700cs model, Agilent Technologies, Tokyo, Japan). Samples were ablated using a 75 × 75 µm laser beam, a 55 μm s^−1^ scan speed, a 20 Hz laser shot repetition rate and a laser fluency (energy) of ca. 3.5 J cm^−2^. The laser aerosol was transported with 800 mL He.min^−1^ toward the ICP MS where ^194^Pt, ^195^Pt and the internal standard (^115^In) were detected at each pixel position in the sample. Homemade software using the Python programming language (www.python.org) was used to generate images of element concentrations per pixel using a color code.

## Results

### Patients

Six patients were included in the study with a median age of 57.7 years (range 51–68), and all patients were in menopausal hormonal status. Four of them were treated for colorectal adenocarcinoma (ADK), and two patients were treated for low-grade appendiceal mucinous neoplasm (LAMN). The patients treated for ADK received at least one oxaliplatin-based FOLFOX induction chemotherapy prior to HIPEC. No other known associated disease was reported for all patients. Patients’ characteristics and treatments are listed in Table 1.

**Table 1:** Clinical data of patients and oxaliplatin-based treatment.

Patient acronym	Age, years	Pathology origin	Peritoneal carcinomatosis index (PCI)	Neoadjuvant chemotherapy (cycle number)	HIPEC [oxaliplatin], mg/m^2^
P1	51	Colorectal adenocarcinoma	0	FOLFOX	Oxaliplatin 250
P2	59	Low-grade appendiceal mucinous neoplasm	0	None	Oxaliplatin 250
P3	53	Colorectal adenocarcinoma	2	FOLFOX	Oxaliplatin 250
P4	63	Colorectal adenocarcinoma with hepatic metastasis	16	FOLFOX (×2) TOMOX (×4)	Oxaliplatin 250
P5	52	Low-grade appendiceal mucinous neoplasm	13	None	Oxaliplatin 460
P6	68	Colorectal adenocarcinoma	3	FOLFOX (×3)	Oxaliplatin 460

PCI, peritoneal cancer index according to Jacquet [26]; HIPEC, hyperthermic intraperitoneal chemotherapy.

### Clinical features of ovaries

Histological analyses of the set of slices taken from the ovaries of six patients did not show metastatic lesions. These were post-menopausal ovaries with a flattened surface epithelium, a cortex with different levels of stromal proliferation that comprised a dense subepithelial network rich in reticulin and collagen (albuginea), albican bodies and a highly vascularized medullary. The studied slices revealed a cortex of variable thickness in relation to the stromal proliferation and a 200 µm thick tunica albuginea. Neither pathological features nor difference in the stromal density were noted for patients with a history of systemic chemotherapy compared with the rest of the group.

### MALDI-MS imaging

**Figure 1:**
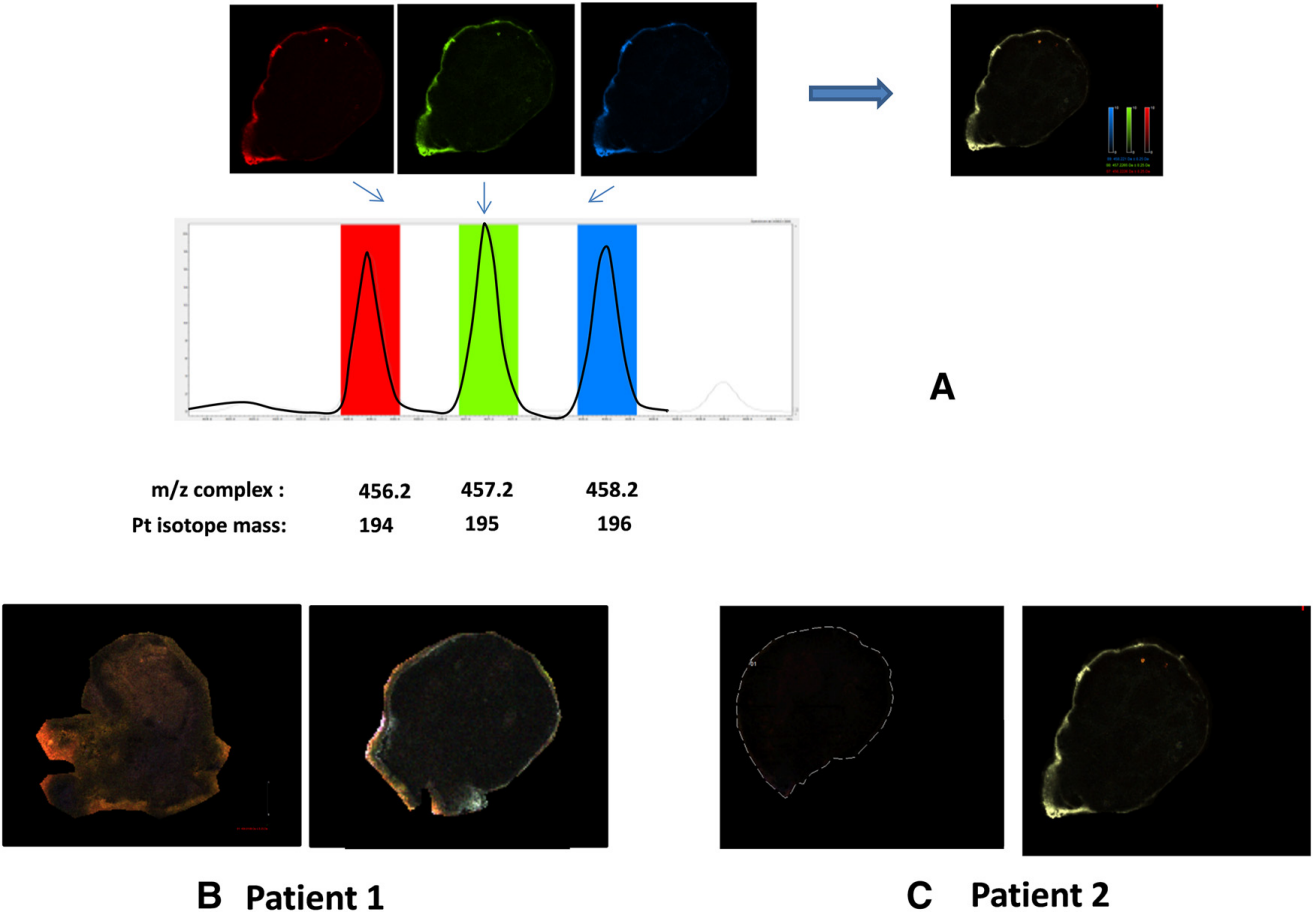
MALDI-MS images. (A) Identification by MALDI mass spectrum of isotope-specific platinum compounds in tissues at *m*/*z* 456.2, 457.2, and 458.2, which correspond to platinum isotopes 194, 195, and 196. The presence of platinum is demonstrated by the exact co-localization of the masses of the three signals from each platinum isotope as illustrated by the merged resulting picture (right panel). Panels (B) and (C): localization of an oxaliplatin complex in a slice of ovaries from patients P1 and P2 before (left) and after HIPEC (right).

We analyzed ovaries collected from patients P1 and P2. Both received the same HIPEC protocol (250 mg/m^2^), but patient P1 received systemic FOLFOX chemotherapy before surgery. The MALDI-MS images (Figure 1) revealed the distribution of a platinum complex, which was evidenced by the triplet mass signature at *m*/*z* 456.21, 457.21, and 458.21 (Figure 1A), which corresponded to ions comprising the 194, 195, and 196 platinum isotopes, respectively. The exact co-localization of their masses and the abundance of the isotopic ratio demonstrated that this mass spectrum signal issued from a platinum-containing compound. The platinum-containing entity did not penetrate the tissue of the tunica albuginea, as shown in the ovaries from patients P1 and P2 (Figure 1B, C, right). No platinum derivative was detected in the contralateral ovary resected before HIPEC (Figure 1B, C, left). Our analytical conditions revealed no other platinum signature (mass triplet) for the tissue, which means that no other form of platinum compound was detected using these MALDI ionization conditions.

### LA-ICP MS imaging

**Figure 2:**
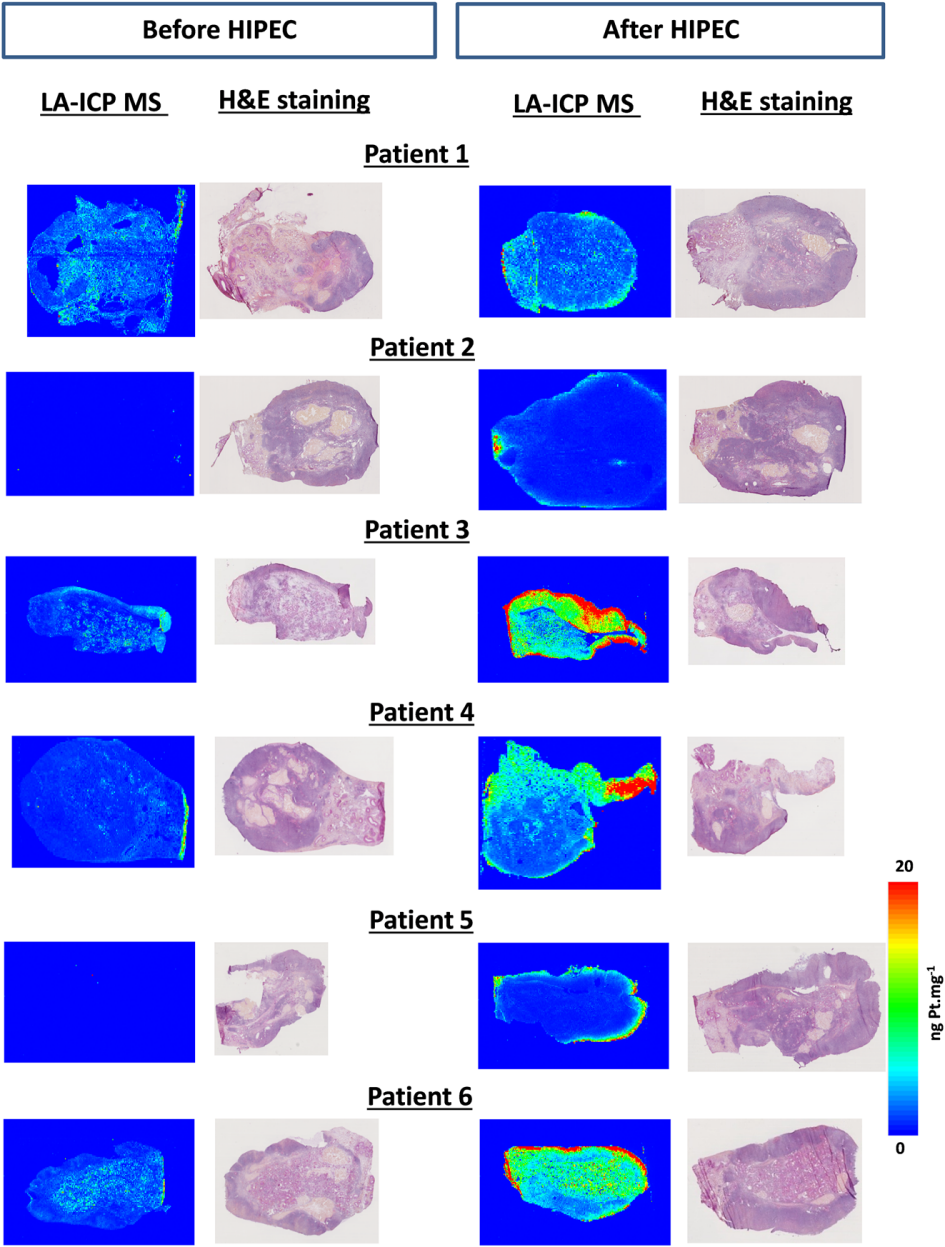
LA-ICP MS quantitative images of cryosections of the ovaries of the six patients. For each patient, the LA-ICP MS localization of elemental platinum (^195^Pt) is illustrated before (left panel) and after (right panel) HIPEC. For each tissue, the hematoxylin/eosin staining of the immediate adjacent slice is presented at the right of the LA-ICP MS picture. Note that no platinum was detected before HIPEC in the ovaries of patients P2 and P5. Color scale on the right from 0 to 20 ng mg^−1^ Pt applies to all pictures. Note that for patient P2 after HIPEC treatment, the signal (of ca. 500 ng mg^−1^) in the core of the ovarian elemental image is due to an analytical artifact.

Tissues sections from the ovaries of six patients collected before and after HIPEC were analyzed using LA-ICP MS imaging. Figure 2 shows that the LA-ICP MS imaging of the ovaries confirmed the findings from the MALDI-MS imaging that elemental platinum after HIPEC was localized in the tunica albuginea of all patients’ ovaries in concentrations ranging from 5 to greater than 20 ng mg^−1^ per pixel. Notably, LA-ICP MS imaging also revealed the presence of platinum compounds deeper in the ovary structure of patients after HIPEC. The concentration in the internal structures was estimated at 4–10 ng mg^−1^ after HIPEC (P1, P3, P4, P6), but remained undetectable in patients P2 and P5 (Figure 2, right panel). Elemental platinum was also detected as traces (<2 ng mg^−1^) in ovaries collected before HIPEC in patients P1, P3, P4, and P6.

## Discussion

Women who are about to undergo PM treatment have various options when it comes to fertility preservation, such as embryo, oocytes as well as ovarian tissue cryopreservation [28], [29]. However in some case, these options need to delay the chemotherapy or surgery and could compromise the success of the treatment. In such case, the direct fertility preservation during the chemotherapy must be considered. The present study is the first study to examine the ovarian penetration of hyperthermic IP oxaliplatin at doses of 250 or 460 mg/m^2^ for an exposure time of 30 min and to consider fertility preservation in women of childbearing age with low-grade neoplasia. An international survey in 2011 collected data of pregnancies in women who underwent CRS with HIPEC and adnexal preservation [14]. Only seven cases of spontaneous pregnancies were reported after HIPEC using cisplatin or mitomycin C as the cytotoxic agent. Five of them were treated for PMP, and two cases were treated for peritoneal mesothelioma [14]. To our knowledge, no cases of pregnancy were communicated after HIPEC when oxaliplatin was used as the cytotoxic drug. The use of oxaliplatin and higher concentrations may be toxic to the ovaries that are not covered by the peritoneum and come in direct contact with the chemotherapy solution. Systematic adnexectomy may be questioned in cases of young women of childbearing age with low-grade neoplasia and low PCI. To make the best choice between the preservation of fertility and the possible extension of the disease to the preserved ovary [30], it is necessary to evaluate the impact of cytotoxic IP treatment on the ovary itself.

The detection and localization of drugs or metallic atoms in human tissues requires sensitive and resolutive imaging techniques, such as MALDI MS [24, 31, 32] or LA-ICP MS [25, 33, 34]. LA-ICP MS was used to localize elemental platinum in animal [35] and human tissues [25]. Our previous studies on the distribution of oxaliplatin-derived entities in tissues revealed that the drug penetrated only a few millimeters in animal tissue [24] and human PM nodules resected after the HIPEC procedure [25].

The present study used a mass spectrometry imaging approach to study possible “off-target” effects of the HIPEC protocol due to the diffusion of the platinum drug into an organ, the ovary, which is not the target of the therapy. The MALDI-MS images showed the distribution of a platinum entity. Although this platinum-containing molecule had the same nominal mass as methionine-oxaliplatin, this finding does not guarantee its identity because its structure must be studied using a more accurate method, such as Fourier-transform ion cyclotron resonance (FTICR). As previously observed for other tissues [24], [25], this platinum-containing entity did not penetrate the tissue deeper than the tunica albuginea, as shown in the ovaries from patients P1 and P2.

LA-ICP MS imaging confirmed the findings of MALDI-MS imaging of the presence of platinum at the periphery of the ovaries from patients P1 and P2 after HIPEC. However, the detection of Pt derivatives in MALDI is strictly dependent on the ionization conditions, and the adjustment of many instrumental parameters is required for each imaged molecule because there is no generic protocol for analysis [31]. Although LA-ICP MS does not allow the determination of a chemical structure, its ionization mode (i.e., atomization of the platinum molecule first then ionization of platinum) enables a quantitative analysis via the detection of all platinum present in the sample. Therefore, the impregnation of a tissue by platinum derivatives may be assessed even for those hardly or not ionized in MALDI-MS and consequently not detected. Therefore, LA-ICP MS imaging provided a more complete picture of the platinum distribution inside the thin ovary sections, and the gain in instrumental sensitivity (limit of detection of 0.1 ng mg^−1^) and the quantitative data acquisition provided essential information. LA-ICP MS imaging detected Pt in all ovaries resected after HIPEC and showed that Pt penetrated more deeply than revealed by MALDI-MS. Platinum was detected in various concentrations ranging from 0 to 5 ng mg^−1^ in patients one, two, and five and from 0 to 20 ng mg^−1^ for patients three, four, and six. Our data showed that the distribution of platinum and its concentration were independent of the concentration of the chemotherapeutic bath. However, more intriguing was the difference in the Pt distribution in the ovaries of patients who received an intravenous platinum-based chemotherapy before HIPEC (P1, P3, P4, and P6) from patients who did not (P2, P5). Whereas Pt was detected in ovaries from patients that received previous adjuvant platinum chemotherapy, no Pt was detected before HIPEC in the ovaries of two patients who did not received this therapy. For these last two patients, the localization of Pt after HIPEC was restricted to the surrounding structure of the ovaries.

The history of platinum-based chemotherapy prior HIPEC was clearly evidenced by the Pt impregnation of the ovaries before HIPEC. In the ovaries of patients who received an intravenous oxaliplatin infusion, Pt was mostly distributed in the medulla before HIPEC because this substructure contains the blood vessels and nerves, and it is more irrigated by the blood flow than the tunica albuginea. Notably, platinum was not detected in the cortex just beneath the tunica albuginea. After HIPEC, Pt compounds were concentrated in the tunica albuginea and the cortex to a lesser extent for patients P1, P2, P4 and P5, but it was distributed more deeply in the medulla of the ovaries for patients who received prior adjuvant platinum chemotherapy than patients who did not receive treatment. The quantitative analysis showed that the penetration of Pt in ovaries during HIPEC was greatly enhanced by the systemic treatment, except for in patient P1, in whom this effect was less obvious. In the ovaries of patients P3, P4, and P6, the Pt concentration increased from 2 to 5 ng mg^−1^ before HIPEC to 10–20 ng mg^−1^ after HIPEC. Elemental Pt was not detected in the most blood-irrigated structure of ovary of patients that did not received systemic treatment, suggesting that the hematogeneous distribution of the drug during HIPEC remains minimal at the end of the time of the intervention.

The platinum-based chemotherapy protocol FOLFOX (5-fluorouracil, leucovorin, oxaliplatin) administered prior HIPEC was clearly associated with a deeper Pt impregnation of the ovaries. In spite of the relatively small number of patients included in the study, the magnitude of the difference in Pt concentration in the deep areas of the ovary, which rises from undetectable in patients who did not receive systemic chemotherapy to a value as high as 20 ng mg^−1^ in those who did receive it, allows us to formulate an hypothesis but does not exclude random effects. From patient P4, for whom we had a complete treatment history, we observed that Pt persisted in the ovary at least one year after the systemic administration.

Few biological functions are concentrated in the tunica albuginea, where the Pt concentrated during the HIPEC of patients who did not receive a systemic treatment. We hypothesized that the HIPEC impact may be minimal on ovarian functions in these conditions. The deeper platinum penetration in the medulla that followed systemic treatments appeared to enhance the diffusion of platinum compounds in this structure during HIPEC. Finally, except for patient three, platinum penetration was less deep in the cortex, which consists of a very cellular connective tissue stroma in which the ovarian follicles are embedded. The observed potentiation of the drug penetration by intravenous systemic oxaliplatin-based chemotherapy may be considered in the decision to preserve fertility during the HIPEC treatment of the peritoneal extension of CRC.

## Conclusions

We used mass spectrometry imaging and demonstrated the oxaliplatin derivative penetration into the ovaries of women treated with HIPEC for a PM extension of digestive cancers. LA-ICP MS imaging demonstrated the distribution of elemental platinum with greater sensitivity than MALDI-MS imaging, which made it possible to propose a molecular nominal mass for the oxaliplatinum complex. Notably, we found that the penetration into ovaries during HIPEC was strongly enhanced when patients received a systemic oxaliplatin-based chemotherapy before HIPEC. The relative preservation of the cortex vs. platinum penetration in patients that didn’t receive a systemic platinum based chemotherapy suggests that the functional properties of the ovaries may not be affected, but further functional studies are needed. PMP is the pathology that meets the combination of these conditions. The effects of systemic therapy preceding HIPEC on the penetration of the drug into targeted tissues and off-target organs must be more generally addressed to prevent side effects and enhance the tumor response to the treatment.
